# Exposure to Second Hand Tobacco Smoke among 12 year old Adolescents in Mangalore, Karnataka - A Descriptive Study

**DOI:** 10.31557/APJCP.2021.22.3.827

**Published:** 2021-03

**Authors:** Ashwini Rao, Unnikrishnan B, Nikita Rungta, Nandini M, Ramya Shenoy, Arathi Rao, Mranali K Shetty

**Affiliations:** 1 *Manipal College of Dental Sciences, Mangalore, A Constituent Unit of Manipal Academy of Higher Education (MAHE), Manipal, India. *; 2 *Kasturba Medical College, Mangalore, A Constituent Unit of Manipal Academy of Higher Education (MAHE), Manipal, India. *; 3 *Department of Biochemistry, Kasturba Medical College, Mangalore, A constituent unit of Manipal Academy of Higher Education (MAHE), Manipal, India. *

**Keywords:** Adolescent, attitude, environmental exposure, knowledge, second hand tobacco smoke

## Abstract

**Background:**

There is no safe level of exposure to second hand tobacco smoke (SHS). The World Health Organization has stressed that 100% smoke-free environments are the only effective way to protect the population from the harmful effects of exposure to SHS.

**Methods:**

A descriptive cross-sectional questionnaire study was done on 1442, 12 year old, adolescents in Mangalore to determine the exposure to SHS and adolescents’ knowledge, attitude, avoidance and self-efficacy of avoidance towards SHS.

**Results:**

The percentage of children exposed to SHS at home was 28.6%. A higher number of male students reported that their parent and/or sibling smoked tobacco compared to their female counterparts. About 48% of the participants reported that persons who lived with them smoked in front of children and this was found to be significantly higher among males compared to females. Atleast 46% of the participants had knowledge of second hand tobacco smoke. The avoidance behaviour of the participants was good with most of the participants reporting positive avoidance towards SHS. With respect to their self - efficacy of avoidance of SHS, most of them were confident of avoiding SHS when they were with family or friends but the confidence was less with respect to strangers. Multivariate general linear model analysis showed a significant association between gender and exposure to SHS to 14 items out of the 25 items in the four domains. Males and those not exposed to SHS showed better knowledge, positive attitude, positive avoidance behaviour and positive self efficacy of avoidance to SHS.

**Conclusion:**

The findings of our study indicate that better knowledge and a positive attitude and avoidance behavior are associated with reduced exposure to SHS and this reinforces the fact that a sustained health education program incorporated into the school curriculum is the need of the hour.

## Introduction

“*Tobacco is the single most preventable cause of death in the world today. During the 21st century, it is estimated to kill one billion*” (WHO, 2000)

Although many countries of the World have passed laws making workplaces, public places, and restaurants smoke-free, still millions of children and adults continue to breathe second hand tobacco smoke (SHS) (CDC, 2007). According to the World Health Organization (2020), the health of almost half of the world’s children is affected by exposure to environmental tobacco smoke (ETS). SHS exposure has been linked to a variety of serious diseases in adults as well as in children, such as coronary heart disease, lung cancer, breast cancer, respiratory symptoms and illnesses, otitis media and during pregnancy causes pre-term low birth weight deliveries (WHO, 2020). Exposure to tobacco smoke in early childhood may result in the development of behavioral problems too (Wada et al., 2020). There is no safe level of exposure to second hand smoke. Even brief exposures can be harmful. Moreover, studies (Farkas et al., 2000; Öberg et al., 2011 ) have even shown that “*children exposed to SHS are more likely to become smokers themselves when they grow older compared to those unexposed*”. 

The US Environmental Protection Agency has classified tobacco smoke as a known human carcinogen, with no safe level of exposure. Inhaling tobacco smoke is the main source of nicotine exposure in the general population (CDC, 2017). Cotinine, a major metabolite of Nicotine is a useful biomarker with a half - life of about 16 to 20 hours and can be used to distinguish tobacco users from non- users (CDC, 2007). Cigarettes contain about 1.5% nicotine by weight, which produces 1–2 mg of bioavailable nicotine per cigarette. 

Legislations banning smoking in enclosed public places have been widely introduced, with a reported 109 countries around the World having implemented legislations (WHO, 2012). In India, beginning with the Cigarettes Act, 1975, many legislations have been implemented to reduce tobacco use. The Cigarette and Other Tobacco Products Act was introduced in 2003, and the Framework Convention on Tobacco Control brought into force in 2005 (GOI, 2003). The Government of India adopted the legislation for banning smoking in public places in 2008 (Mehrotra et al., 2010). These legislations have given some hope to the general population that the tobacco menace might one day see the end that it envisages for the habituer. However, legislations do not cover smoking inside homes. It has been found that children are especially at risk of SHS exposure at home since they spend a lot of time in close proximity to their parents (Adgate et al., 2004; Matt et al., 2008). This led to the conceptualization of the present study among adolescents in Mangalore, India, with the aim of assessing their exposure to second hand tobacco smoke and to determine their knowledge, attitude, avoidance behaviour and self - efficacy of avoidance towards second hand tobacco smoke. 

## Materials and Methods

This study is the descriptive component of the multiphase study conducted among 12 year old school children of Mangalore, India (Rao et al., 2019). A cluster random sampling of all the schools in Mangalore was done to obtain the required sample. A questionnaire was then administered to the 12 year olds in the selected schools, who fulfilled the inclusion criteria. 

*Sample size calculation*

The sample size estimation was done using the formula: 

With a design effect at 1.5 and a non-response at 5%, the final sample size was calculated to be 1400.

*Study procedure*

After obtaining approval from the Block Education Officer, a cluster random sampling of all the schools in Mangalore was done to obtain the required sample size of 1,400. A questionnaire was then administered to the 12 year olds in the selected schools, who fulfilled the inclusion criteria, i.e., who consented to participate in the study and those participants whose parents gave written informed consent and they themselves give informed assent. 

*Data collection*

Demographic data was recorded. The questionnaire had two components, one to assess children’s exposure to SHS using 5 items (Lin et al., 2010). The second component was a 25 item questionnaire (Gharaibeh et al., 2011) to determine Children’s knowledge, attitude avoidance behaviour and self-efficacy of avoidance towards second hand smoke. Knowledge was assessed using 10 items, Attitude with 5 items, Avoidance behaviour towards second hand smoke was assessed using 5 items and Self-efficacy of avoidance using 5 items. Knowledge refers to the understanding of the participants towards tobacco and SHS, attitude refers to their feelings towards SHS, avoidance behaviour is the action taken to avoid SHS and self efficacy of avoidance is their confidence in regulating avoidance behaviour.

*Reliability of the questionnaire*

The questionnaire was administered to 10 individuals and a Cronbach’s coefficient alpha of 0.8 was obtained indicating high reliability. 

*Statistical analysis*

Analysis was done based on the Intention to Treat analysis. The data was entered into the SPSS software (IBM Corp. Released 2011. IBM SPSS Statistics for Windows, Version 20.0. Armonk, NY: IBM Corp.) and analysed. Descriptive statistics are presented wherever necessary. Chi square test was done for categorical variables. Multivariate general linear model analysis was done with gender and exposure to SHS at home as the dependent variable and the items and domains of the questionnaire as the independent variables. The level of significance was kept at 0.05 with 95% confidence levels. 

## Results

The questionnaire was administered to 1,460 children and we got a total of 1,442 completed questionnaires back. Among them there was almost equal representation of gender with 722 participants being males and 720 females. 

*Exposure to SHS*

When the participants were asked if anyone at home smoked tobacco, 236 children (16.4%) reported that somebody in their house smoked tobacco and 22.5% reported that those who visited their house smoked tobacco. About 10.8% of the adolescents (155), reported that their parent smoked, 7 (0.5%) reported that their siblings smoked and 63 (4.4%) said that someone else who lived with them smoked tobacco. Eleven participants also reported more than one person smoking tobacco at home. More males reported exposure to SHS at home compared to females and it was statistically significant. When we compared the responses, we found that more male students reported that their parent and/or sibling smoked tobacco compared to their female counterparts. However, more females (52.4%) reported that somebody else they lived with smoked tobacco compared to males (47.6%). This was found to be statistically significant (P=0.036). About 22% of the participants also reported that outsiders who visited them, smoked at home and thus the total participants exposed to SHS at home rose to 28.6%.(Table 1). 

The next item was to find out if people had specified designated places to smoke at home. We found that 140 participants (59.3%) out of the 236, reported that those who stayed at home smoked only outside the house. Although 11 participants reported having a designated room for smoking in the house, 85 participants said that people who stayed with them smoked anywhere they wanted inside the house (Table 2).

Out of the people 324 participants (22.5%) who reported that outsiders who visited them, smoked at home, 238 (73.5%) of them said that they smoked outside the house, whereas 62 reported that they smoked anywhere inside the house and 24 in one room only. More males reported that people who visited their house smoked anywhere inside the house, as compared to females and it was statistically significant (Table 3).About 48% (114) of the participants reported that the persons who live with them smoked in front of children whereas only 13.3% of participants reported that people who visit them smoke in front of children. Smoking in front of children was found to be significantly higher among males compared to females (Table 4).

*Knowledge Domain*

Only 46% of the participants knew that Second-hand tobacco smoke is generated from the side-stream of a cigarette or from the cigarette smoke puffed out by smokers. More numbers of male participants (408) had knowledge of this as compared to the female participants (257) and the difference was statistically significant. (P<0.0001) (Table 5). Only 57.8% of the participants felt that as long as they did not smoke, long-term exposure to second-hand tobacco smoke would not be harmful to their health. The number of males who believed that, was higher than the females and it was found to be statistically significant. (P<0.0001). About 50% of the participants did not know that a smouldering cigarette was more toxic than the smoke that was exhaled by a smoker. When we compared males and females, we found that less number of females (274) knew this compared to the males (374) and this was found to be statistically significant (P<0.0001). A total of 667 (46.3%) felt that even if not actively smoking, one has to worry about the damage to one’s health that may be caused from second-hand tobacco smoke. About 67% of the participants knew that when a parent is a current smoker, the child has a higher risk for developing lung cancer. More number of males (528) knew this compared to females (444). This was also found to be statistically significant. About 57% knew that a lit cigarette burning in an ashtray would affect the health of people nearby. A higher number of males (468) had knowledge of this compared to females (359). This was also found to be statistically significant. 

Only about 54% of the participants knew that long-term second-hand tobacco smoke affects the lungs and the heart. The remaining 46% thought that it affects only the lungs but not the heart. This difference was also found to be statistically significant. Among the participants only 56% knew that long-term second-hand tobacco smoke is responsible for lung cancer in non-smokers and the knowledge among males was statistically higher than among the females. About 62% knew that smoking was not allowed in train, airplane and also in cars. The number of females who reported knowledge of this was lesser than that of males. This was found to be statistically significant. About 60% of the participants knew that second-hand tobacco smoke is a toxic cocktail consisting of cancer producing chemicals. The knowledge was statistically higher among males compared to females (Table 5).

*Attitude Domain*

Only about 5% of the participants thought that it was worthwhile to take the initiative to avoid passive tobacco smoke in order to protect one’s health. Among them, more number of females showed this attitude compared to males and it was found to be statistically significant. About 76% of the participants reported that they need to pay constant attention to the avoidance of second-hand tobacco smoke, more among males compared to females, which was found to be statistically significant. When family members or friends smoked at home, 41.7% thought that it was okay to avoid the area where they were smoking, but the remaining 58.3% thought it was impolite. More number of males felt it was okay compared to females and it was found to be statistically significant. Only 7% of the participants felt that it was a troublesome matter when someone smoked beside them. For the rest 93% it was not a troublesome matter. About 72% of the total participants, more so among males than females, felt that when they were in a second-hand smoke environment, by asking smokers not to smoke, or requesting them to smoke somewhere else, they were doing something to protect their health. This was found to be statistically significant (Table 6).

*Avoidance Behavior domain*

About 79% of the participants reported that when someone in their family smoked in front of them, they would choose to leave in order to avoid second-hand smoke. Similarly 76% also reported that they would do the same in public places too. More number of males expressed this behaviour as compared to females and it was found to be statistically significant. About 70% of the participants also felt that if they could not avoid a second-hand smoke environment, they would open the window to ventilate the smoke in the room. When asked if someone smoked in front of them, would they ask him or her to stop smoking or to smoke elsewhere, 76% of the participants reported that they would do so at home and 71% of them said they would do that in public places too (Table 7).

*The Self efficacy of avoidance domain*

Almost 89% of the participants reported having the confidence to request their friends to stop smoking and 87% had the confidence to request family members to stop smoking at home. However only 65% had the confidence to ask strangers not to smoke in banned public spaces. About 74% were confident that they could avoid second-hand smoke while with friends and about 75% were confident that they could avoid second-hand smoke while with relatives or elders (Table 8).

*Questionnaire items based on exposure to SHS*

When we categorized the participants based on their exposure to SHS, there were 413 children who were exposed and 1029 who were not exposed to SHS. We analysed the questionnaire items based on the childrens’ exposure to SHS and we found that among the 10 items of the knowledge domain, 4 items showed a statistically significant difference between those exposed and not exposed to SHS. We found that those who were exposed to SHS had lesser knowledge about SHS compared to those who were not exposed and this was found to be statistically significant (P<0.05) (Table 9). 

When the attitude domain was analysed, we found that those who were not exposed to SHS showed a positive attitude to safeguarding their health with respect to SHS and this was found to be statistically significant among the 3 items of the attitude domain.

When the avoidance behaviour domain was analysed, we found that those who were not exposed to SHS, reported better avoidance behaviour to SHS exposure compared to those exposed and this was found to be statistically significant among the 3 items of the avoidance behavior domain.

In the self efficacy domain too, those who were not exposed to SHS showed a higher confidence towards reaching out to people to minimise SHS exposure (P<0.05). This showed that the participants who reported SHS exposure at home not only had a significantly lower knowledge about SHS, but also did not have a positive attitude, positive avoidance behaviour and positive self efficacy of avoidance towards SHS exposure (Table 9).

When we analysed domain-wise scores gender-wise, we found that knowledge and attitude domains showed a statistically significant difference with males showing positive scores compared to females with respect to knowledge and attitude towards SHS exposure (Table 10). However domain-wise scores did not show a statistically significant difference when we compared those exposed and those not exposed to SHS.

*Association between gender, exposure to SHS at home and the items of the questionnaire*

When multivariate general linear model analysis was done with gender and exposure to SHS at home as the dependent variable and the items of the questionnaire as the independent variables, we found that gender was significantly associated with 3 items of the knowledge domain where males were found to be associated with better knowledge about SHS than females. We also found that SHS exposure was significantly associated with 5 items of the knowledge items, where better knowledge was associated with not being exposed to SHS. In the attitude domain, we found that an statistically significant association existed between SHS exposure and two items and those who reported no exposure to SHS were found to have a more positive attitude towards preventing SHS exposure compared to those who reported to be exposed to SHS. In the domain of avoidance behaviour, three items showed a statistically significant association with gender, with males reporting better avoidance behaviour compared to females and one item showed an association with SHS exposure, wherein those participants not exposed to SHS were confident of asking someone to stop smoking or to smoke elsewhere, if they found them smoking in public places. In the domain of self efficacy of avoidance domain, we found gender to be significant associated with 2 items, with boys showing better confidence in asking strangers to not smoke in public places whereas girls showed better confidence in avoiding SHS while at home with relatives and elders (Table 11).

When multivariate general linear model analysis was done with gender and exposure to SHS at home as the dependent variable and the domains of the questionnaire as the independent variables, we found that knowledge domain was significantly associated with both gender and SHS exposure, with males and those not exposed to SHS being associated with better knowledge about SHS compared to females and those exposed to SHS. The domain of avoidance behaviour was also significantly associated with SHS exposure, indicating that those with no exposure to SHS showed better avoidance behaviour domain scores compared to those who were exposed to SHS (Table 12).

**Table 1:** Exposure to SHS from Those Who Stay at Home

	Males	Females	Total
Parent	96 (61.9)	59 (38.1)	155
Sibling	4 (57.1)	3 (42.9)	7
Someone else you live with	30 (47.6)	33 (52.4)	63
More than one person at home	7 (63.6)	4 (36.4)	11
Somebody at home smoked	137 (58.1)	99 (41.9)	236 (16.4)
Outsiders who visit smoked	182 (56.2)	142 (43.8)	324 (22.5)
Total participants exposed to SHS at home	232 (56.2)	181 (43.8)	413 (28.6)

P, 0.036; numbers in parenthesis represent percentages, columns will not add up

**Table 2:** Designated Places to Smoke for Those Who Stay at Home

	Males	Females	Total
Anywhere inside the house	55 (64.7)	30 (35.3)	85
Only in one room	4 (44.4)	7 (55.6)	11
Only outside the house	78 (56.2)	62 (43.8)	140
Total	137 (58.1)	99 (41.9)	236

P, 0.014; numbers in parenthesis represent percentages

**Table 3:** Designated Places to Smoke - for Those Who Visit Home

	Males	Females	Total
Anywhere inside the house	40 (68.8)	22 (31.2)	62
Only in one room	16 (66.7)	8 (33.3)	24
Only outside the house	126 (52.9)	112 (47.1)	238
Total	182(56.2)	142(43.8)	324

**Table 4:** Distribution Based on Smoking in front of Children

	Males	Females	Total	Total smokers	P value
People who live with you smoke in front of children	77 (67.5)	37 (32.5)	114 (48.3)	236	P=0.00
People who visit you smoke in front of children	29 (67.4)	14 (32.6)	43 (13.3)	324	P=0.15

P<0.05, Significant; Numbers in parenthesis represent percentages

**Table 5:** Distribution Based on Knowledge - Gender-Wise

	Males (722)	Females (720)	Total (1442)	P value
Second-hand tobacco smoke is generated from the burning end of a cigarette or from the cigarette smoke puffed out by smokers	408 (56.5)	257 (35.7)	665 (46.1)	0.000*
Even though I do not smoke, long-term exposure to second-hand tobacco smoke will be harmful to my health	464 (64.2)	369 (51.3)	833 (57.8)	0.000*
A smouldering cigarette is more toxic than the smoke that is exhaled by a smoker	374 (51.8)	274 (38.1)	648 (44.9)	0.000*
Even if not actively smoking, one has to worry about the damage to one’s health that may be caused from second-hand tobacco smoke.	368 (51)	299 (41.5)	667 (46.3)	0.000*
If one is a current smoker, one’s child has a higher risk for developing lung cancer	528 (73.1)	444 (61.7)	972 (67.4)	0.000*
A lit cigarette burning in an ashtray will affect the health of people nearby	468 (64.8)	359 (49.9)	827 (57.4)	0.000*
Long-term second-hand tobacco smoke affects the lungs and the heart	424 (58.7)	350 (48.6)	774 (53.7)	0.000*
Long-term second-hand tobacco smoke is responsible for lung cancer in non-smokers.	450 (62.3)	357 (49.6)	807 (56)	0.000*
Not only train and airplane passengers, but even car passengers cannot smoke	461 (63.9)	435 (60.4)	896 (62.1)	0.000*
Second-hand tobacco smoke is a toxic cocktail consisting of cancer producing chemicals	478 (66.2)	385 (53.5)	863 (59.8)	0.000*

*P<0.05, Significant; numbers in parenthesis represent percentages

**Table 6:** Distribution Based on Attitude - Gender-Wise

	Males (722)	Females (720)	Total (1442)	P value
I think it is worthwhile to take the initiative to avoid passive tobacco smoke in order to protect one’s health	34 (4.7)	37 (5.1)	71 (4.9)	0.000*
I think we need to pay constant attention to the avoidance of second-hand tobacco smoke	568 (78.7)	523 (72.6)	1091 (75.7)	0.000*
When family members or friends smoke in the home, I think it is okay to avoid the area where they are smoking	328 (45.4)	274 (38.1)	602 (41.7)	0.001*
Whenever someone smokes beside me, it is a troublesome matter	51 (7.1)	51 (7.1)	102 (7.1)	0.065
When you are in a second-hand smoke environment, by asking smokers not to smoke, or requesting them to smoke somewhere else, you are doing something to protect your health	541 (74.9)	495 (68.8)	1036 (71.8)	0.014*

*P<0.05, Significant; numbers in parenthesis represent percentages

**Table 7:** Distribution of Avoidance Behavior - Gender-Wise

	Males (722)	Females (720)	Total (1442)	P value
In my family, if someone smokes in front of me I will choose to leave in order to avoid the second-hand smoke	593 (82.1)	549 (76.3)	1142 (79.2)	0.000*
In public places when people smoke in front of me, I will choose to leave in order to avoid the second-hand smoke	563 (78)	539 (74.9)	1102 (76.4)	0.018*
When I cannot avoid a second-hand smoke environment, I will open the window to ventilate the smoke in the room	513 (71.1)	493 (68.5)	1006 (69.8)	0.045*
In my home, if someone smokes in front of me I will ask him or her to stop smoking or I will ask him or her to smoke elsewhere	545 (75.6)	549 (76.3)	1094 (75.9)	0.185
In public places, if someone smokes beside me I will ask him or her to stop smoking, or I will ask him or her to smoke elsewhere	513 (71.1)	507 (70.4)	1020 (70.7)	0.227

*P<0.05, Significant; numbers in parenthesis represent percentages

**Table 8:** Distribution Based on Self-Efficacy of Avoidance - Gender-Wise

	Males (722)	Females (720)	Total (1442)	P value
I have the confidence to request my friends to stop smoking	635 (88)	642 (89.2)	1277 (88.6)	0.223
I have the confidence to request my family members to stop smoking in the home	619 (85.7)	634 (88.1)	1253 (86.9)	0.054
I have the confidence to ask strangers not to smoke in banned public spaces	507 (70.2)	434 (60.3)	941 (65.3)	0.000*
I am confident that I can avoid second-hand smoke while with friends	539 (74.7)	527 (73.2)	1066 (73.9)	0.000*
I am confident that I can avoid second-hand smoke while with relatives or elders	539 (74.7)	541 (75.1)	1080 (74.9)	0.016*

*P<0.05, Significant; numbers in parenthesis represent percentages

**Table 9:** Distribution of Questionnaire Items –SHS Exposure Wise

	Exposed (413)	Not exposed (1029)	Total (1442)
Knowledge			
If one is a current smoker, one’s child has a higher risk for developing lung cancer	296 (30.5)	676 (69.5%)	972 (67.4%)*
A lit cigarette burning in an ashtray will affect the health of people nearby	216 (26.1)	611 (73.9)	827 (57.4)*
Not only train and airplane passengers, but even car passengers cannot smoke	233 (26.0)	663 (74)	896 (62.1)*
Second-hand tobacco smoke is a toxic cocktail consisting of cancer producing chemicals	222 (25.7)	641 (74.3)	863 (59.8)*
Attitude			
I think it is worthwhile to take the initiative to avoid passive tobacco smoke in order to protect one’s health.	32 (45.1)	39 (54.9)	71 (4.9)*
When family members or friends smoke in the home, I think it is okay to avoid the area where they are smoking.	150 (24.9)	453 (75.1)	603 (41.8)*
Whenever someone smokes beside me, it is a troublesome matter	41 (40.2)	61 (59.8)	102 (7.1)*
Avoidance Behavior			
In public places when people smoke in front of me, I will choose to leave in order to avoid the second-hand smoke	301 (27.3)	801 (72.7)	1102 (76.4)*
In my home, if someone smokes in front of me I will ask him or her to stop smoking or I will ask him or her to smoke elsewhere	297 (27.1)	797 (77.5)	1094 (75.9)*
In public places, if someone smokes beside me I will ask him or her to stop smoking, or I will ask him or her to smoke elsewhere	259 (25.4)	761 (74.6)	1020 (70.7)*
Self efficacy of Avoidance			
I have the confidence to request my friends to stop smoking	354 (27.7)	923 (72.3)	1277 (88.6)*
I have the confidence to ask strangers not to smoke in banned public spaces	250 (26.6)	691 (73.4)	941 (65.3)*

*P<0.05, Significant; numbers in parenthesis represent percentages

**Table 10:** Participants with Positive Scores towards Knowledge – Gender Wise

Domain	Male (722)	Female (720)	Total (1442)	P value
Knowledge	504	384	888	0.000*
Attitude	305	266	571	0.040*
Avoidance Behavior	566	553	1,119	0.47
Self efficacy of avoidance	610	591	1,201	0.221

*P<0.05, Significant

**Table 11:** Multivariate General Linear Model Analysis of Gender and Exposure to SHS at Home and the Items of the Questionnaire

Items of the questionnaire	Dependent Variable	F	P value
Knowledge			
Second-hand tobacco smoke is generated from the burning end of a cigarette or from the cigarette smoke puffed out by smokers	Gender	29.947	0.001
Even though I do not smoke, long-term exposure to second-hand tobacco smoke will be harmful to my health	SHS exposure	4.657	0.031
If one is a current smoker, one’s child has a higher risk for developing lung cancer	SHS exposure	12.533	0.0001
A lit cigarette burning in an ashtray will affect the health of people nearby	Gender	11.629	0.001
	SHS exposure	4.044	0.045
Long-term second-hand tobacco smoke is responsible for lung cancer in non-smokers	SHS exposure	4.282	0.039
Not only train and airplane passengers, but even car passengers cannot smoke	Gender	6.348	0.012
Second-hand tobacco smoke is a toxic cocktail consisting of cancer producing chemicals	SHS exposure	6.566	0.01
Attitude			
I think it is worthwhile to take the initiative to avoid passive tobacco smoke in order to protect one’s health	SHS exposure	7.374	0.007
When family members or friends smoke in the home, I think it is okay to avoid the area where they are smoking.	SHS exposure	4.198	0.041
Avoidance Behavior			
In my family, if someone smokes in front of me I will choose to leave in order to avoid the second-hand smoke	Gender	5.391	0.02
When I cannot avoid a second-hand smoke environment, I will open the window to ventilate the smoke in the room.	Gender	6.199	0.013
In public places, if someone smokes beside me I will ask him or her to stop smoking, or I will ask him or her to smoke elsewhere	SHS exposure	12.426	0.0001
Self efficacy of Avoidance			
I have the confidence to ask strangers not to smoke in banned public spaces	Gender	10.42	0.001
I am confident that I can avoid second-hand smoke while with relatives or elders	Gender	6.473	0.011

*P<0.05, Significant

**Table 12:** Multivariate General Linear Model Analysis of Gender and Exposure to SHS at Home and the Domains of the Questionnaire

Domains	Dependent Variable	F	P value
Knowledge domain	Gender	50.042	0.000*
	SHS Exposure	14.599	0.000*
Attitude domain	Gender	1.387	0.239
	SHS Exposure	0.153	0.696
Avoidance Behavior domain	Gender	0.479	0.489
	SHS Exposure	8.968	0.003*
Self efficacy of avoidance domain	Gender	0.534	0.465
	SHS Exposure	0.861	0.354

*P<0.05, Significant

## Discussion

In the present study, the percentage of children exposed to SHS at home was 28.6% whereas in a study by Ghazali et al., (2019) and Lim et al., (2019) the exposure to SHS among Malaysian adolescents was 41.5% and 50%. Park (2020) in Korea, reported an exposure of 55.1%, which was found to be higher than our study. Precioso et al., (2019) reported that 25.6% of children in Portugal claimed that they were exposed to SHS at home. When we compared the present study with studies done among adolescents in India, we found that the exposure to SHS at home in Kerala (Rakesh et al., 2017) was 23.2% and was 49% in Pondicherry (Arikrishnan et al., 2020), which was higher than our study.

In our study we also found that 59% of the participants reported that those who smoked at home did so only outside the house and 36% smoked anywhere they wanted to inside the house. Mamudu et al., (2015) in their pooled analysis of the nationally representative 2006 to 2009 Global Youth Tobacco Survey data of West Africa found that the SHS exposure inside the home ranged from 13.0% to 45.0% and SHS exposure outside the home ranged from 24.7% to 80.1%.

With respect to the guests visiting the homes, 22.5% were smokers, although it was reported that 73.5% of those guests smoked outside the house. It was noteworthy to find that significantly more number of male participants reported that their parents and guests smoked tobacco, as compared to their female counterparts. About 48% of the participants in our study reported that the persons who lived with them smoked in front of children which was higher compared to the study by Kovess et al., (2013) who reported that 19.3% and 10.0% of Eastern and Western European mothers, respectively, smoked in the vicinity of their children.

When we analysed the questionnaire, we found that atleast 45% of the participants had knowledge of second hand tobacco smoke, which was low compared to the study by Arikrishnan et al., (2020) in Pondicherry where he found that about 70.1% had adequate knowledge about SHS and its harmful effects. Our study also found that the knowledge about SHS was better among males as compared to females, which was in contrast to the findings of Lim et al., (2019) who reported that there was better knowledge and awareness of harmful effect of SHS among female students compared to males, in their study on Malaysian adolescents. 

However, in the attitude domain, the responses of the present study were not very encouraging. Most of the participants (95%) did not think it was worthwhile to take the initiative to avoid passive tobacco smoke in order to protect one’s health and about 93% did not perceive someone smoking beside them as a troublesome matter, which was quite alarming. More than 58% of the participants also thought that it was impolite to avoid the area where family members or friends were smoking. In our study, male participants showed better attitude towards SHS compared to females. The avoidance behaviour of the participants was good with most of the participants reporting positive avoidance towards SHS. An interesting finding was that in a situation where they were exposed to SHS, a significantly higher number of males reported that they would prefer leaving that place but a significantly higher number of females reported that they would prefer opening the window to ventilate the smoke. With respect to their self - efficacy of avoidance of SHS, most of them were confident of avoiding SHS when they were with family or friends but the confidence was less with respect to strangers. Similar findings were reported by Arikrishnan et al., (2020) who reported that although 40.5% of the adolescents reported that they would advise their family members to stop smoking, almost 80% mentioned that they would not react if they saw someone smoking in a public place. 

In our study, multivariate general linear model analysis showed a significant association between gender and exposure to SHS to 14 items out of the 25 items in the four domains. Males and those not exposed to SHS showed better knowledge, positive attitude, positive avoidance behaviour and positive self efficacy of avoidance to SHS. 

In conclusion, tobacco consumption is a huge public health issue and Tobacco consumption in India is continuing to increase despite tobacco control policies (Mohan et al., 2018). The findings of our study indicate that better knowledge and a positive attitude and avoidance behavior are associated with reduced exposure to SHS and this reinforces the fact that sustained health education program incorporated into the school curriculum is the need of the hour to increase awareness and build positive attitudes and develop confidence among our younger generation, with the hope of not only reducing the number of adolescents taking up the tobacco habit, but also help them become a messenger in educating their family and friends about the ill effects of tobacco, thus contributing to the building of a tobacco-free India and World. 

*Limitations*

The study suffers from those limitations inherent in any questionnaire study, the main one being social desirability bias. Some children did not want to express that someone at home smoked tobacco. This could be because of the stigma associated with it and the fact that it would be ridiculed by their peers. We tried to minimise this by assuring the participants that the information would be kept strictly confidential and their privacy maintained.
